# *Rhabdorrhynchus echii* (Brahm, 1790), a “forgotten” name (Coleoptera, Curculionidae, Lixinae)

**DOI:** 10.3897/zookeys.243.3976

**Published:** 2012-11-16

**Authors:** Massimo Meregalli, Miguel Angel Alonso-Zarazaga

**Affiliations:** 1 Department of Life Sciences and Systems Biology, University of Torino, Via Accademia Albertina 13, 10123 Torino, Italy; 2 Departamento de Biodiversidad y Biología Evolutiva, Museo Nacional de Ciencias Naturales (CSIC), José Gutiérrez Abascal, 2, E-28006 Madrid, Spain

**Keywords:** Neotype, new combination, nomenclature, European fauna, Palaearctic weevils

## Abstract

The application of the name *Curculio echii* Brahm, 1790 is discussed. Based on the description it is evident that it should be applied to a German species of the genus *Rhabdorrhynchus*, and that it has priority over the name currently applied to the species, *Rhabdorrhynchus seriegranosus* Chevrolat, 1873. The new combination *Rhab-dorrhynchus echii* (Brahm, 1790) is proposed. As there is a lack of any type material of *Curculio echii* a neotype is designated. Based on the study of the type specimen, *Rhabdorrhynchus seriegranosus* is restored as a valid species.

## Introduction

In the course of the preparation of the Catalogue of the Palaearctic Coleoptera: Lixinae: Cleonini several nomenclatural questions had to be resolved, and several previously undetected cases of priority came to light. Some of these concerned taxa seldom, if ever, cited in the literature. However, in some cases the “lost” names were applied to taxa more broadly known.

Brahm (1790) published an “Insect Calendar”, in which he mentioned, month by month, the species that he had seen or collected in the surroundings of Mainz (Germany). In most cases he referred them to previously named taxa, but some of the species were described as new. One of these is *Curculio echii* Brahm, 1790: 175. The description is rather accurate, cites the plant where Brahm collected one specimen (*Echium vulgare* L.) and emphasizes its key traits. This description allows identification of *Curculio echii* as belonging to the genus *Rhabdorrhynchus* Motschulsky, 1860. Only one species of the genus is known to occur in Germany, thus the application of the name is undisputed. The comb. n. *Rhabdorrhynchus echii* (Brahm, 1790) is here proposed, based on *Curculio echii* Brahm, 1790: 175.

However, even though this was the first name to have been assigned to this species, it was born under an unlucky star, and it soon became forgotten. The name *Curculio echii*, published as it was in a book that did not have any influence in nomenclature, was not used in subsequent works. It was occasionally cited in nomenclatural checklists, both older ones (Sherborn 1902) and recent on-line name indexes (i.e., ION 2012), but it was never positively applied to any taxon and no transfer to other genera was ever proposed.

In 1795 Herbst described *Curculio varius* Herbst, 1795, from Europe. Regardless to the fact that this name is a junior homonym of *Curculio varius* Fabricius, 1775, and several other senior homonyms, it was continuously applied to the central European species, firstly (Schoenherr 1826) as *Pachycerus varius*, and subsequently, after publication of Chevrolat (1873), as *Rhabdorrhynchus varius*. Chevrolat (1873) also described *Rhabdorrhynchus seriegranosus* Chevrolat, 1873, from Algeria. This name was placed under synonymy of *Rhabdorrhynchus varius* by Faust (1904).

Throughout the 20th century the species, in Faust’s concept, was named *Rhab-dorrhynchus varius* (Csiki 1934, as in all other papers which cited the species, including Ter-Minasian 1988). It ranges in central-southern Europe, northern Africa and western Asia. Eventually, Alonso-Zarazaga and Lyal (1999) discovered the homonymy of *Curculio varius* Herbst, 1795 and its only synonym, *Rhabdorrhynchus seriegranosus* Chevrolat, 1873, became the valid name and it was applied to specimens from Europe, Northern Africa and Western Asia.

However, *Rhabdorrhynchus echii* is the first available name to be applied to the taxon and must be used as the correct name for this species. *Rhabdorrhynchus seriegranosus* was seldom used since 1999 and article 23.9.1.2 ICZN cannot be applied.

**Figure 1–2.**
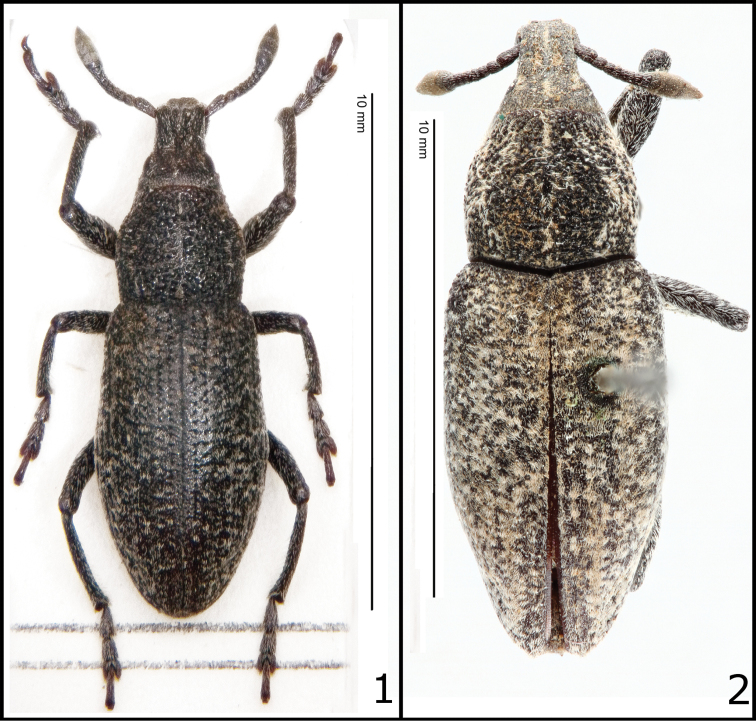
**1** *Rhabdorrhynchus echii* (Brahm, 1790). Neotype, dorsal view **2** *Rhabdorrhynchus seriegranosus* Chevrolat, 1873. Type specimen, dorsal view. Conserved at the Stockholm Museum of Natural History, Chevrolat colletion.

**Figure 3–4.**
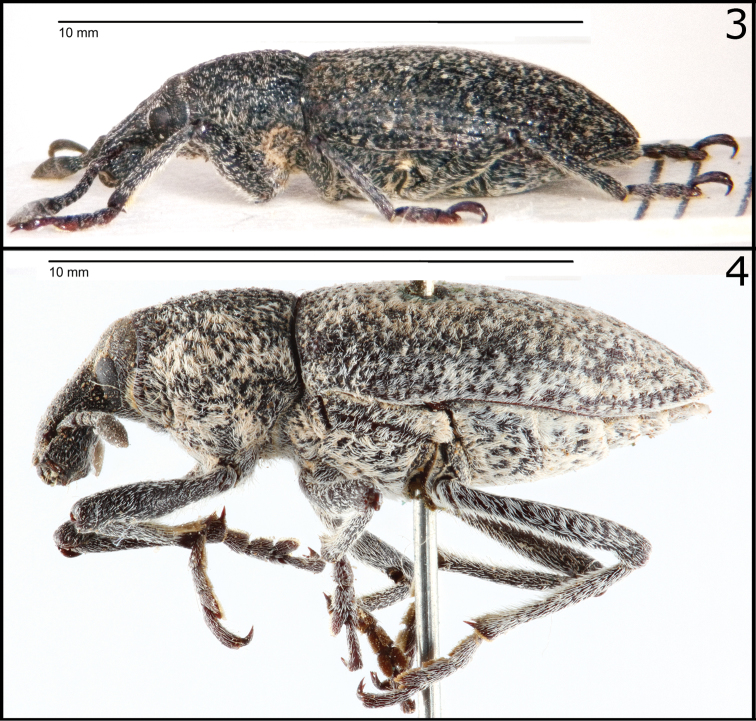
**3** *Rhabdorrhynchus echii* (Brahm, 1790). Neotype, lateral view **4** *Rhabdorrhynchus seriegranosus* Chevrolat, 1873. Type specimen. Same specimen as Fig. 2.

It should be added that the taxonomy of the genus was never thoroughly revised, and there is still uncertainty regarding the validity of some species and their range. The type specimen of *Rhabdorrhynchus seriegranosus*, recently examined by one of the authors (M.M.), differs quite significantly from the European taxon (Figs 1-4), and attribution of specimens from the southern Mediterranean coasts to the central European species, as originally proposed by Faust (1904) and never subsequently discussed, seems questionable. The synonymy *Rhabdorrhynchus echii* (Brahm, 1790) = *Rhabdorrhynchus seriegranosus* Chevrolat, 1873 is here rejected and *Rhabdorrhynchus seriegranosus* is restored as a valid species.

This act also allows maintainance as valid the first epithet attributed to the Algerian *Rhabdorrhynchus*. Two more species were in fact subsequently described from Algeria, namely, *Rhabdorrhynchus curvirostris* Solari, 1950 and *Rhabdorrhynchus longicollis* Solari, 1950, both based on a single specimen, never recollected anywhere else and thus apparently endemic to the country – which is at least unlikely. The status of these species, and their relations with *Rhabdorrhynchus seriegranosus* and the other north-african taxa of the genus, were never discussed after their description.

It is evident that a typification of *Curculio echii* Brahm is required. Nikolaus Joseph Brahm (1751–1821) was a German zoologist, but there is no information about the fate of his collection, which was never cited in the literature. Horn et al. (1990) do not even report the name. Thus we consider the type specimen of *Curculio echii* to be lost.

We hereby designate a neotype of *Curculio echii* Brahm, using a specimen from southern Germany. This act is done with the intent to fix once and for all the meaning of the name and to stabilize nomenclature (under Art. 75.3 of the International Code of Zoological Nomenclature), with particular regard to the central European taxon.

A specimen conserved at the Staatliches Museum für Naturkunde, Stuttgart, Germany, collected in Baden-Württenberg, southern Germany – thus not far from Mainz – is appropriate to be used as the neotype of *Rhabdorrhynchus echii*. An appropriate description for the central-European populations was given by Dieckmann (1983), under *Rhabdorrhynchus varius* Herbst.

*Curculio echii* Brahm 1790. NEOTYPE (here designated): A male specimen so labeled: 1. Germany, Ba- / den–Württ. / Grißheim / 6.Aug.1994 / leg. Kasper // Rheinaue // Rhabdorhyn- / chus varius / Herbst / det. Kasper // *Curculio echii* Brahm 1790 / NEOTYPUS / 2012 Meregalli & Alonso-Z. des. [red]

The range of the species is here considered to include only the forms morphologically referable to *Rhabdorrhynchus echii*, mainly present in Central and central-eastern Europe. The populations from southern Europe, northern Africa and Western Asia are referred to *Rhabdorrhynchus seriegranosus*.
